# The molecular dialogue between the tumor cells and fibroblasts

**DOI:** 10.18632/oncotarget.28405

**Published:** 2023-05-19

**Authors:** Ramesh Butti, Gopal C. Kundu

**Keywords:** CAFs, heterogeneity, myofibroblasts, drug resistance, osteopontin

**Figure 1:**
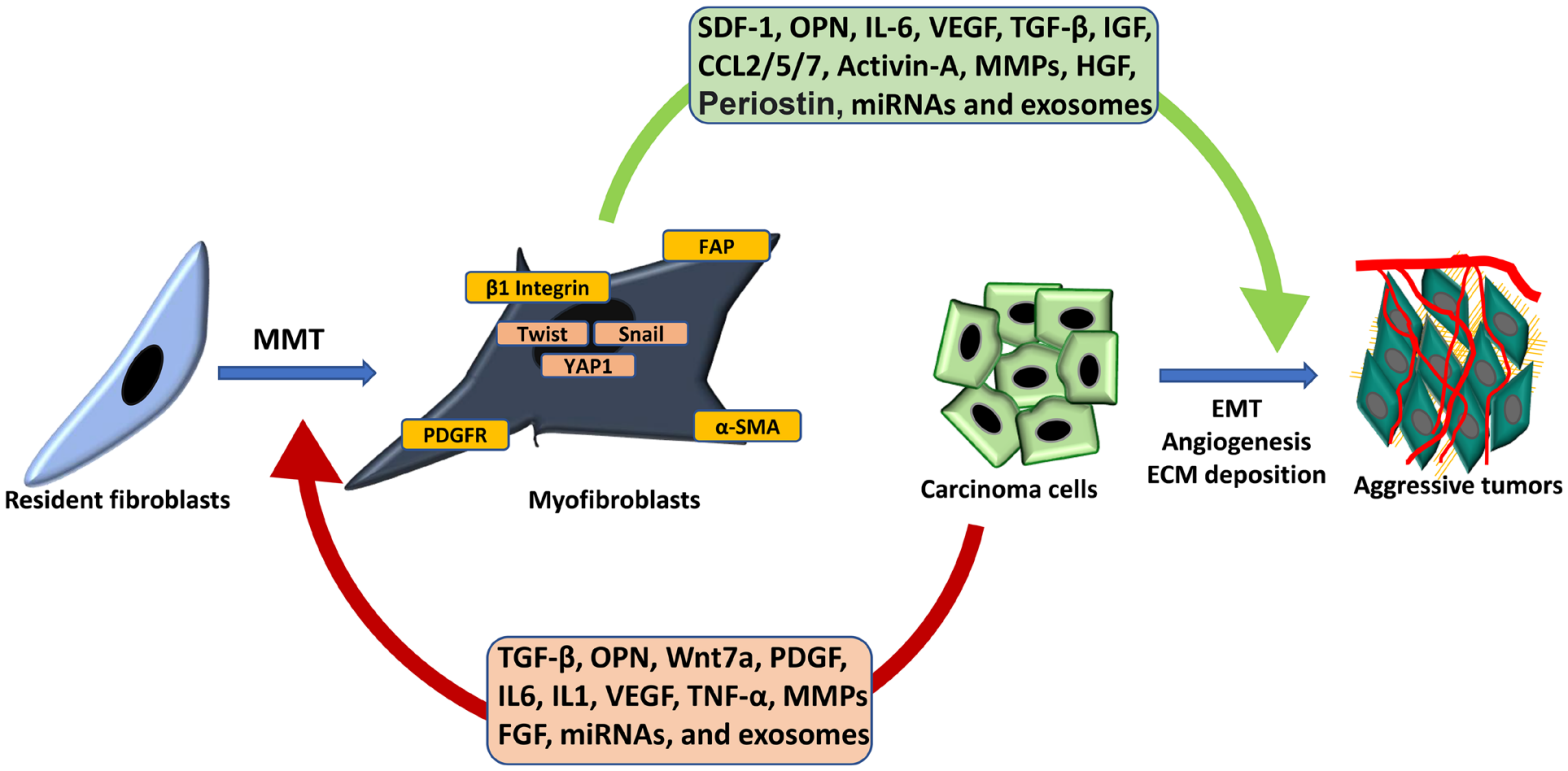
Reciprocal crosstalk between CAFs and cancer cells. Cancer cell-derived factor is involved in the transition of resident fibroblasts to CAFs while CAF-derived factors induce EMT, angiogenesis and ECM deposition in tumors.

The Cancer-associated fibroblasts (CAF) are abundant cell types, present in stromal that are found in stromal pockets of solid tumors. Under normal physiological conditions, fibroblasts exist as quiescent and less migratory cell types with limited synthetic and metabolic activities [1]. However, the fibroblasts which are educated by tumor cells show higher contractability, migratory ability, and ECM deposition [1, 2]. CAFs also secrete array of growth factors and chemokines which have the potential to influence the hallmarks of cancer [3]. The “dynamic interplay” between the cancer cells and fibroblasts is imperative for coevolution of tumor and CAFs [4]. The several growth factors and chemokines are implicated in “mutual crosstalk” of CAFs and cancer cells (Figure 1).

CAFs are highly versatile and plastic populations with both molecular and functional heterogeneity [5]. CAFs have been classified into different subtypes based on the expression of different markers. α-SMA-expressing CAFs are subsets of CAFs referred as myofibroblastic CAFs (myCAFs or myoCAFs) [5, 6]. Several studies demonstrated the generations of myofibroblasts majorly from trans-differentiation of the resident fibroblasts by molecular cues derived from cancer cells. Tumor-borne TGF-β is one of important factors responsible for trans-differentiation of fibroblasts to myofibroblasts [7]. We have also demonstrated similar trans-differentiation program for the CAFs orchestrated by tumor cell-derived Osteopontin (OPN) in breast cancer. OPN induces the expression of Twist1 which in turn drives the expression of myofibroblastic genes [2]. Similar type of CAF gene regulation has also been achieved by IL6 in gastric fibroblasts [8]. OPN-educated CAFs also secrete CXCL12 (SDF-1) [2]. CXCL12 is a very important chemokine implicated in cancer cell growth, inflammation and immune suppression. The CAF-secreted CXCL12 induces EMT in the cancer cells to increase their migratory and angiogenetic potentials [2]. CAFs also secrete IL-6 which in turn augments the expression of OPN in cancer cells to enhance cancer cell proliferation and motility in head and neck cancer model [9]. CAFs are also involved in collagen deposition, thereby hindering the drug delivery and immune cell filtration to impact anti-tumor functions of cancer therapeutics [7]. Because of their crucial role in cancer progression, several CAF-targeting therapies are under clinical trials for the management of cancer [6]. However, recent studies which focused on targeting these cells have challenged this dogma and reported subsets of these cells with tumor restricting functions [10]. Notably, depletion of CAFs lead to accelerated tumor progression with poor disease outcomes in pancreatic cancer [11]. Deletion of collagen type I in myCAFs or targeting of pathways responsible for the generation of myCAFs also led to similar results [10, 12]. These studies highlight the subsets of CAFs with tumor restriction functions. Targeting a subset of CAFs also led to enrichment of others, suggesting “pushful dynamics” among subsets of CAFs [13].

Comprehensive analysis of CAFs using single cell genomics and transcriptomics may shed light on molecular and functional heterogeneity of CAFs. Recent single cell transcriptomics identified several subsets of CAFs depending on types of tumors and position within tumors. However, the study of the conservation of these populations across the species and tissue of origin can elucidate commonalities that may have therapeutic potential. In addition, comprehensive dissecting of the signaling pathways responsible for instigating CAF-mediated tumor progression may facilitate the targeting tumor promoting cues and sparing the inhibitory cues for increasing the efficacy of cancer therapies.
